# Supplementary material for: Semi-Synthesis of C-Ring Cyclopropyl Analogues of Fraxinellone and Their Insecticidal Activity Against Mythimna separata Walker
Source: Molecules. 2020 Mar 2;25(5):1109. doi: 10.3390/molecules25051109 (PMC7179169; doi:10.3390/molecules25051109)

Supplementary Materials

Semi-synthesis of C-ring Cyclopropyl Analogues of Fraxinellone and Their Insecticidal Activity against *Mythimna separata* Walker

Xiao-Jun Yang [1], Qing-Miao Dong [2], Min-Ran Wang [2] and Jiang-Jiang Tang [2,*]

[1] School of Chemistry & Chemical Engineering, Yanan University; yangxiaojun2002@126.com (X.J.Y.)
[2] Shaanxi Key Laboratory of Natural Products & Chemical Biology, College of Chemistry & Pharmacy, Northwest A&F University; qingmiaodong@nwafu.edu.cn (Q.M.D.); 13833683803@163.com (M.R.W.)
* Correspondence: tangjiang11@nwafu.edu.cn (J.J.T.); Tel.: +86-2987-09-2662 (J.J.T.)

List of Contents:

Page S2. **Table S1**. Crystal data and structure refinement for **3a**.

Pages S3-S7. Figure S1-S5 NMR spectra of compounds **2b**, **2c** and **3a-3c**.

Pages S8-S9. Figure S6-S8 HR-ESI-MS spectra of compounds **3a-3c**.

Table 1. Crystal data and structure refinement for **3a**.

Identification code	**3a**
Empirical formula	$C_{23}H_{24}O_5$
Formula weight	380.42
Temperature/K	293(2)
Space group	$P2_12_12_1$
a/Å	8.2081(4)
b/Å	14.3113(8)
c/Å	16.7802(9)
α/°	90.00
β/°	90.00
γ/°	90.00
Volume/Å^3	1971.15(18)
Z	4
ρ_{calc}g/cm^3	1.282
μ/mm^{-1}	0.731
F(000)	808.0
Crystal size/mm^3	0.35 × 0.13 × 0.10
Radiation	CuKα (λ = 1.54178)
2Θ range for data collection/°	8.12 to 132.04
Index ranges	-9 ≤ h ≤ 4, -15 ≤ k ≤ 16, -19 ≤ l ≤ 13
Reflections collected	4353
Independent reflections	2992 [R_{int} = 0.0224, R_{sigma} = N/A]
Data/restraints/parameters	2992/0/257
Goodness-of-fit on F^2	1.081
Final R indexes [I>=2σ (I)]	R_1 = 0.0434, wR_2 = 0.1001
Final R indexes [all data]	R_1 = 0.0568, wR_2 = 0.1097
Largest diff. peak/hole / e Å^{-3}	0.10/-0.12
Flack parameter	0.0(3)

Figure S1. ^{1}H and ^{13}C NMR (MeOD) spectra of compound **2b**.

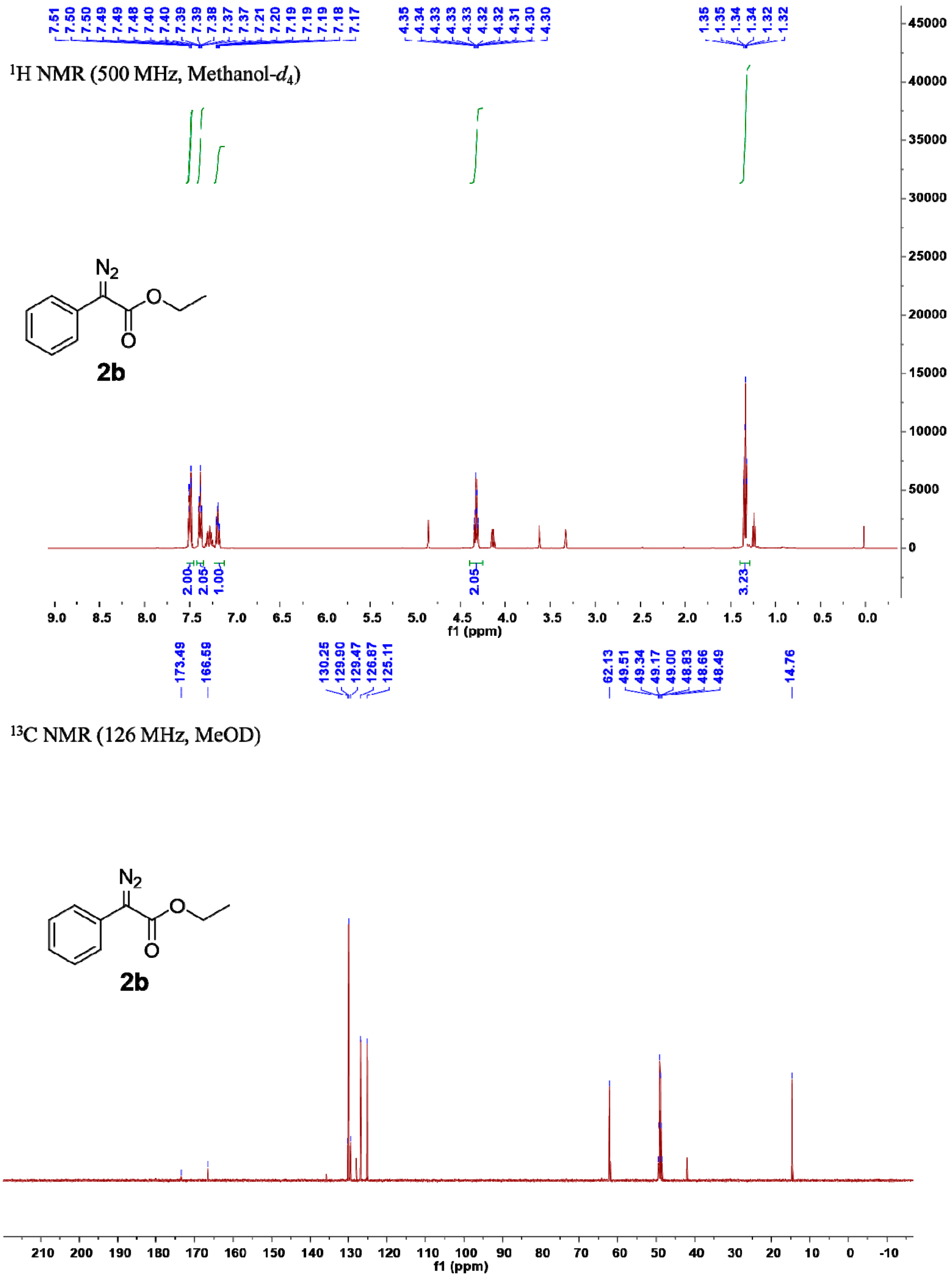

Figure S2. ^{1}H and ^{13}C NMR ($CDCl_3$) spectra of compound **2c**.

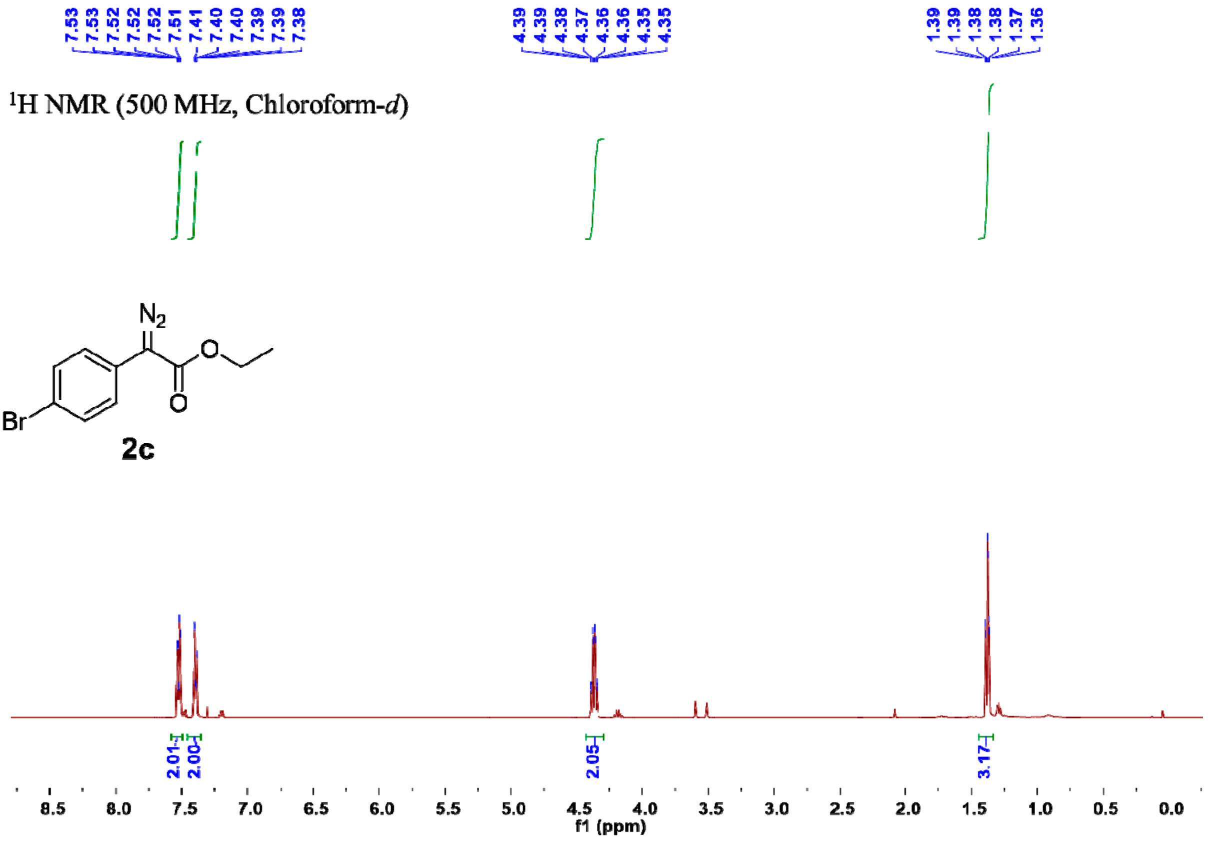

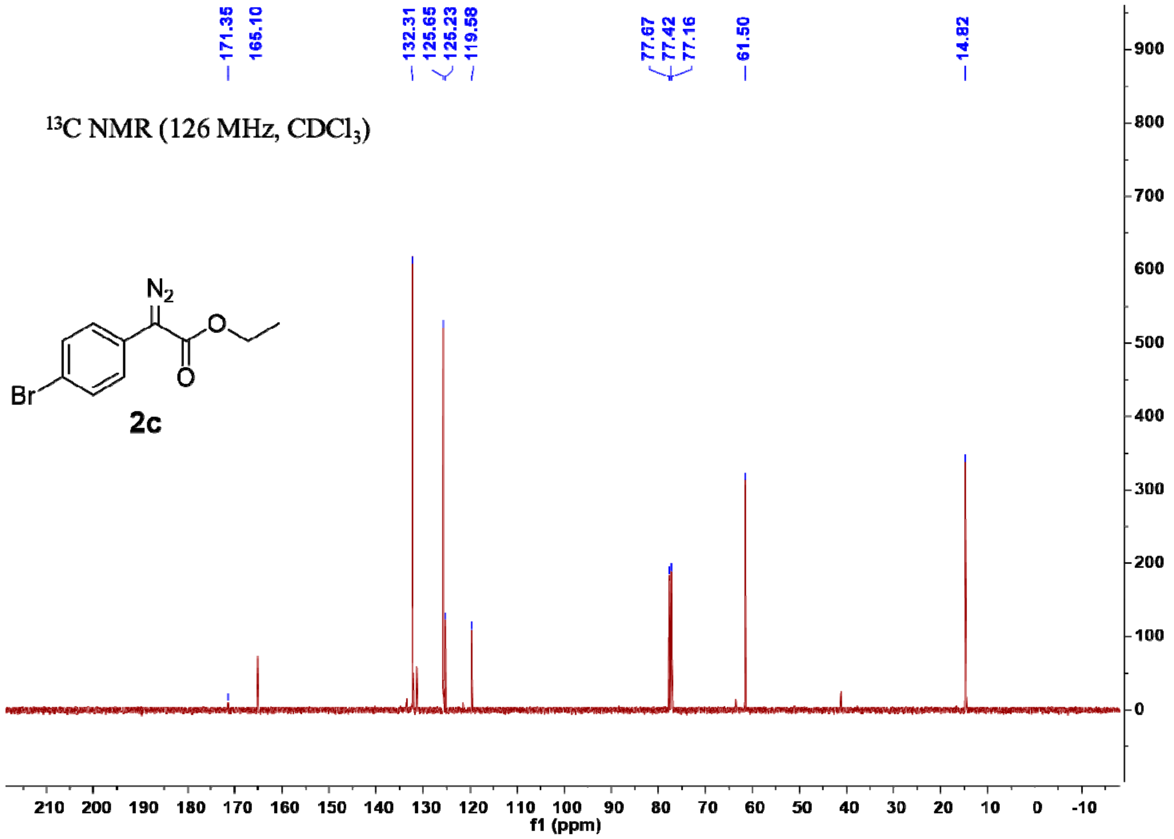

Figure S3. ^{1}H, DEPT135 and ^{13}C NMR ($CDCl_3$) spectra of compound **3a**.

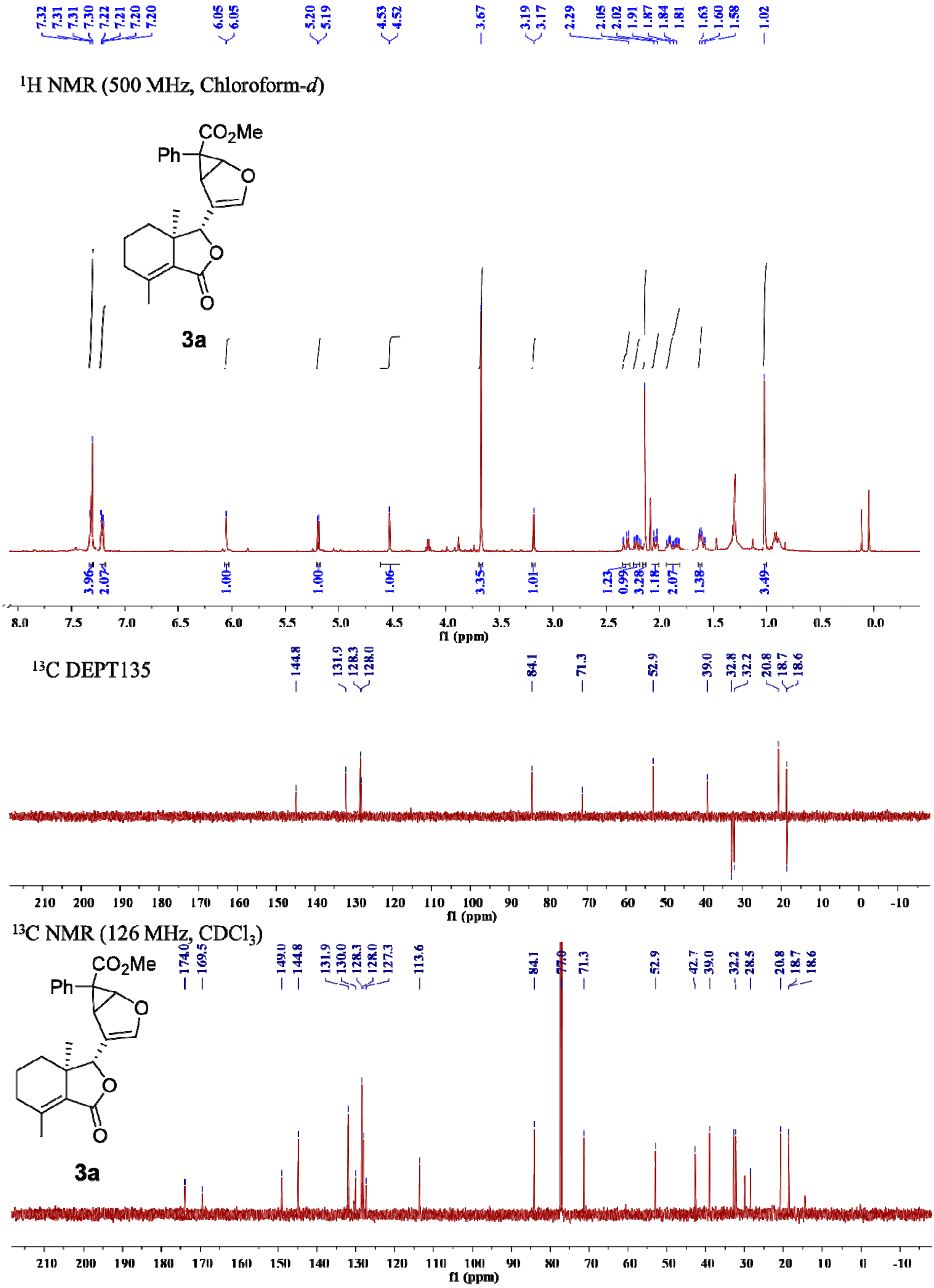

Figure S4. ^{1}H, DEPT135 and ^{13}C NMR ($CDCl_3$) spectra of compound **3b**.

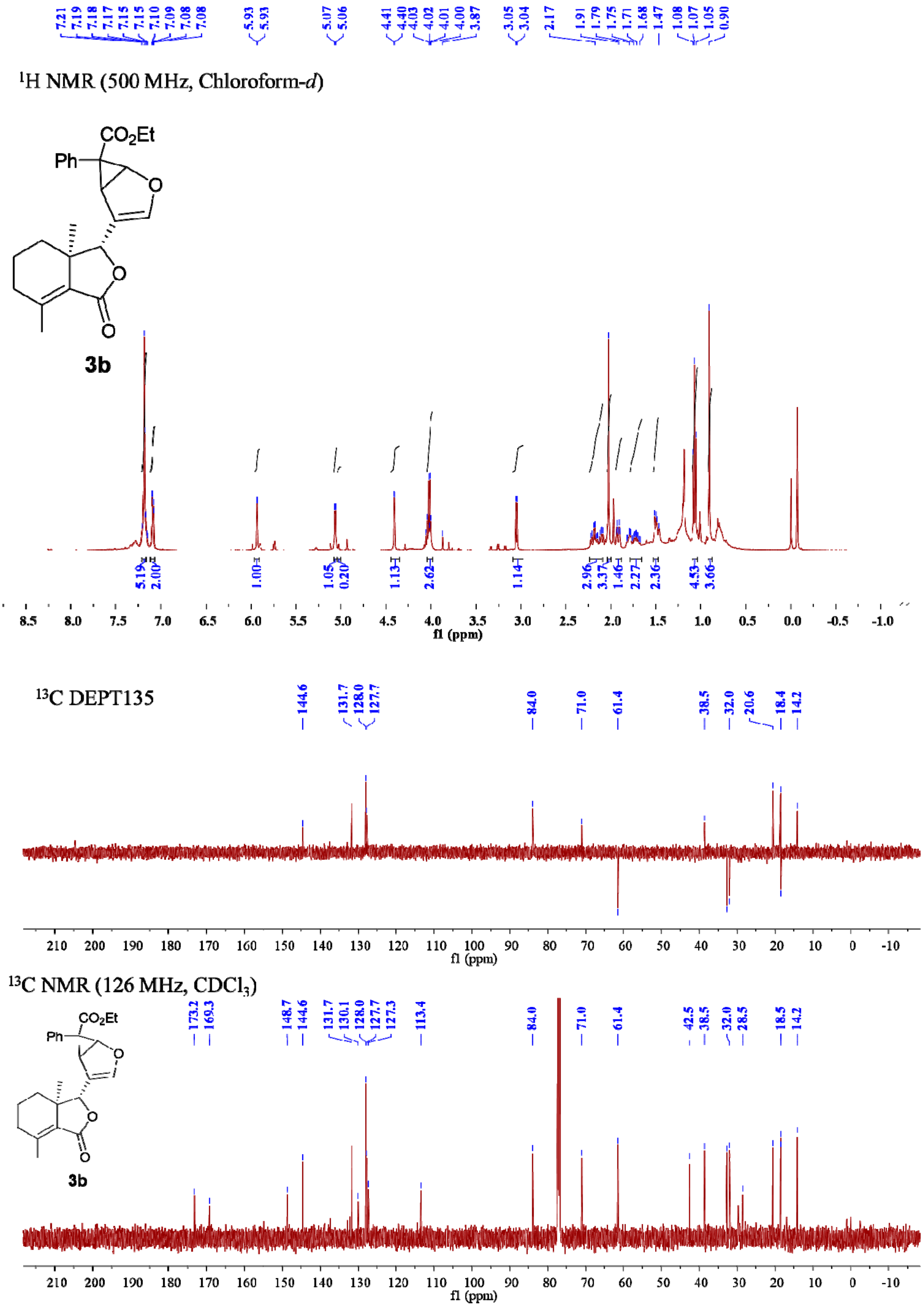

Figure S5. ^{1}H, DEPT135 and ^{13}C NMR ($CDCl_3$) spectra of compound **3c**.

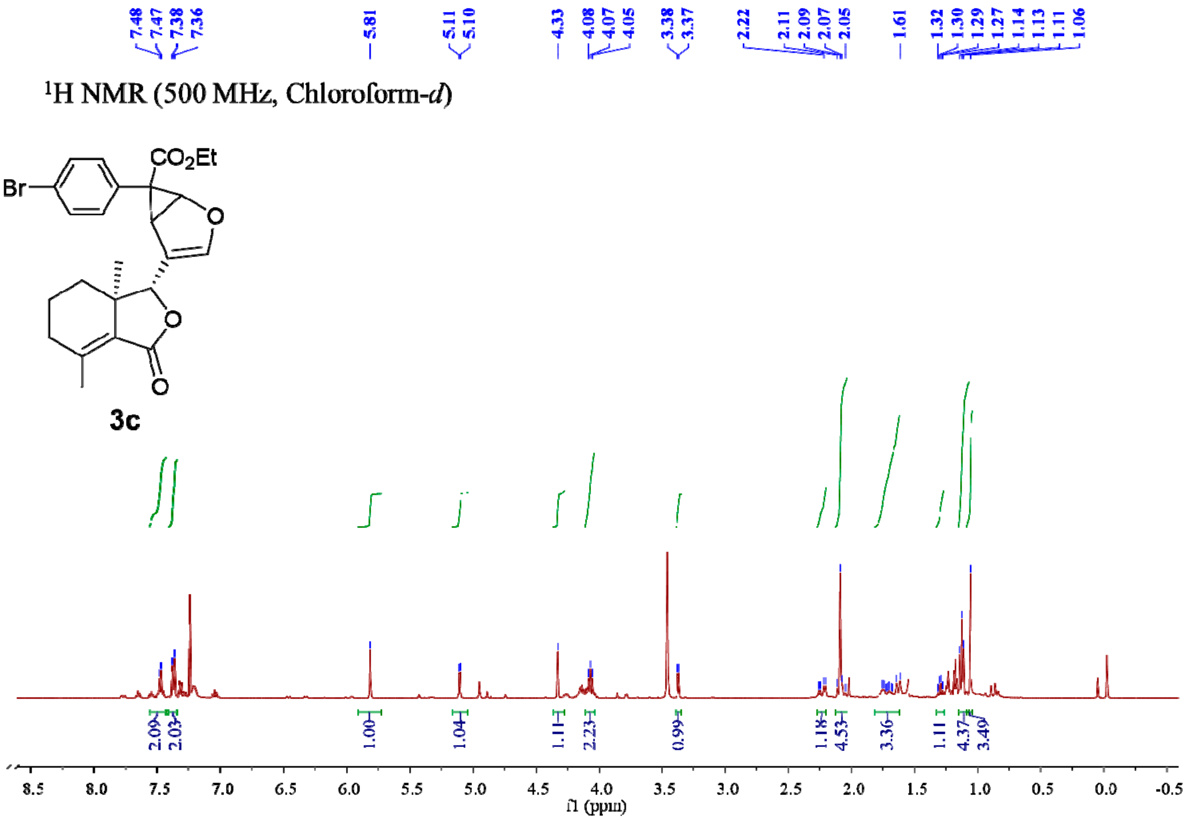

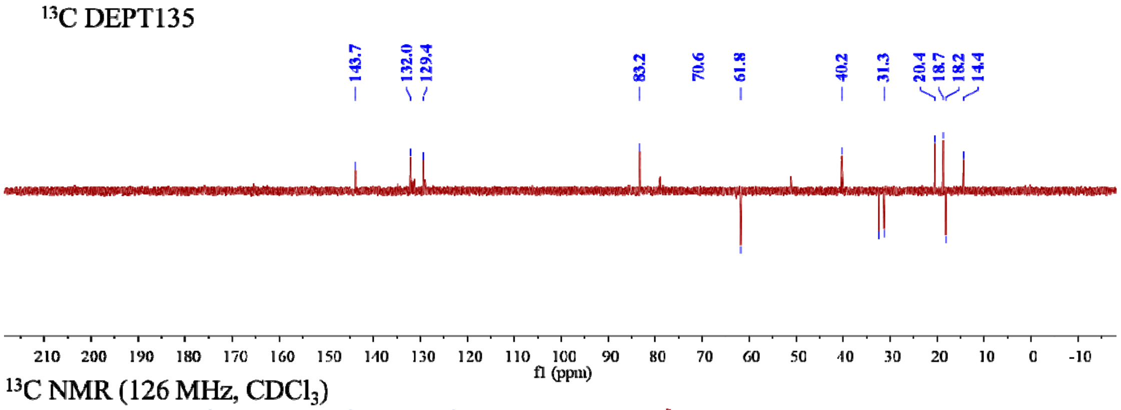

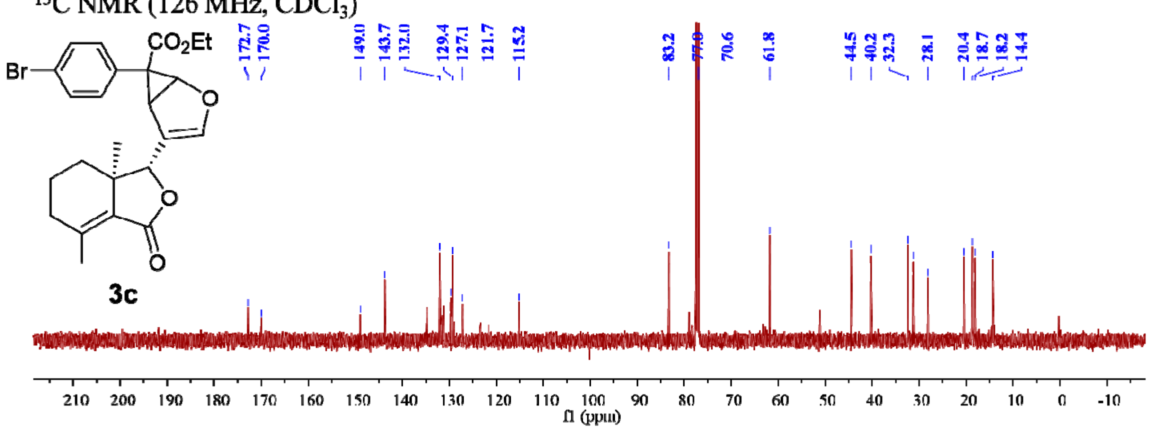

Figure S6. HR-ESI-MS spectra of compound **3a**.

+TOF MS *m/z*: Found 403.1496 $[M+Na]^+$ (calcd for $C_{23}H_{24}NaO_5$, 403.1521).

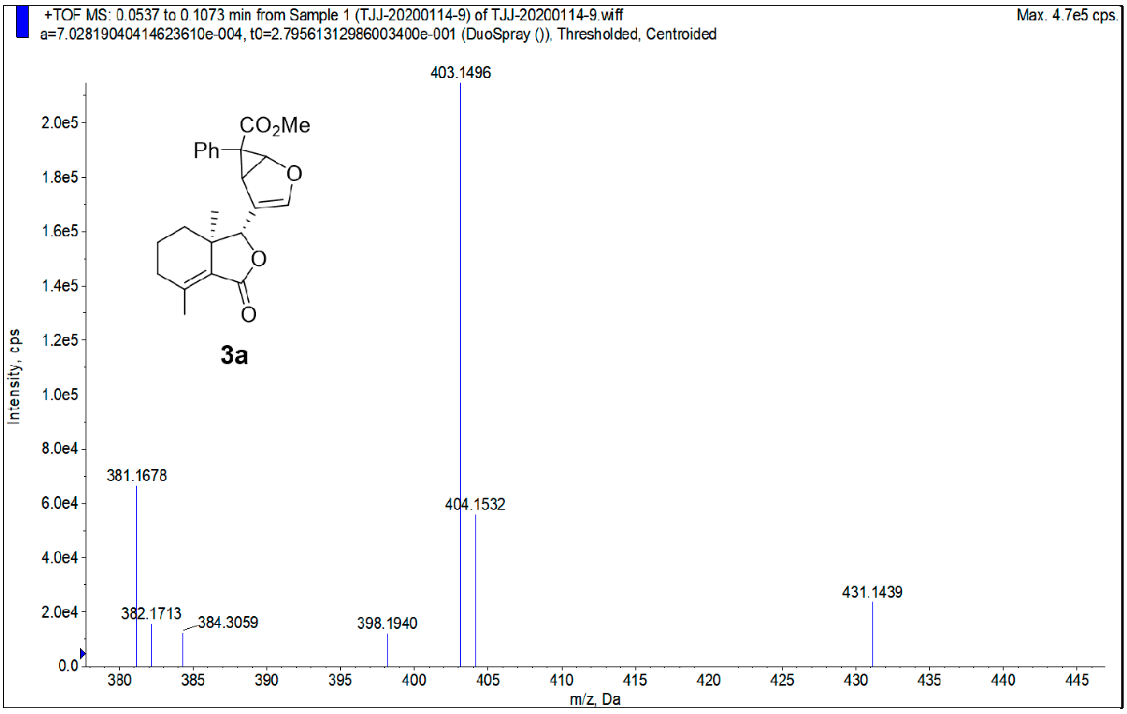

Figure S7. HR-ESI-MS spectra of compound **3b**.

+TOF MS *m/z*: Found 417.1647 $[M+Na]^+$ (calcd for $C_{24}H_{26}NaO_5$, 417.1678).

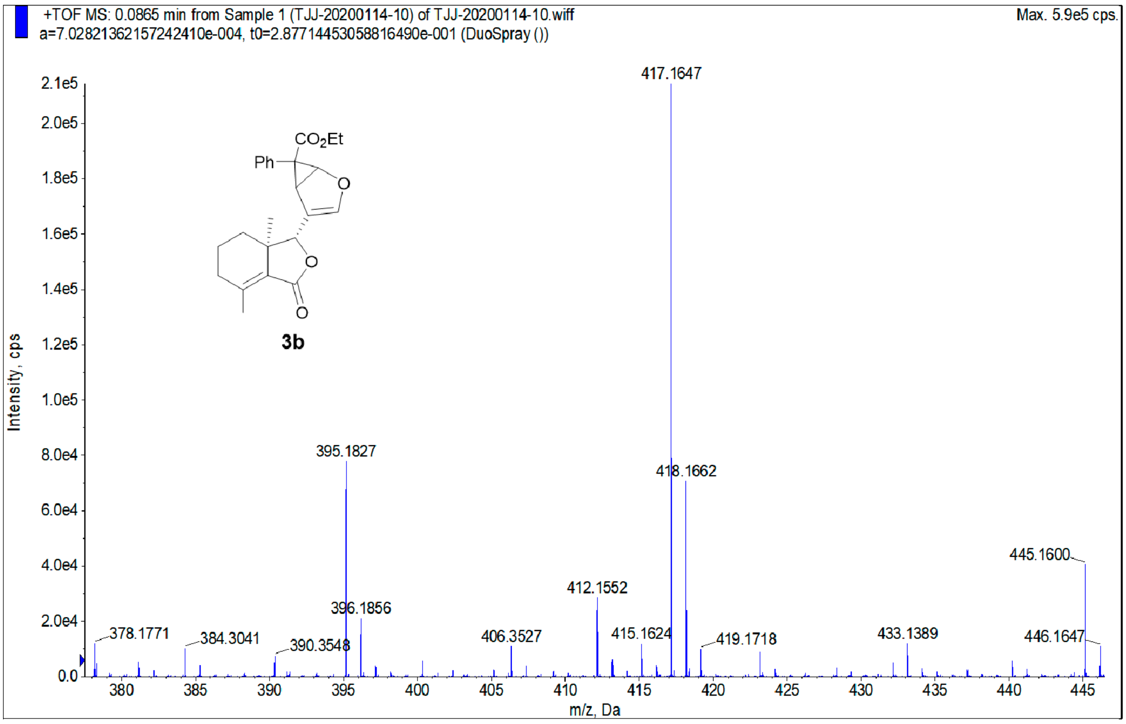

Figure S8. HR-ESI-MS spectra of compound **3c**.

+TOF MS *m/z*: Found 495.0762 [M+Na]+ (calcd for $C_{24}H_{25}BrNaO_5$, 495.0783).

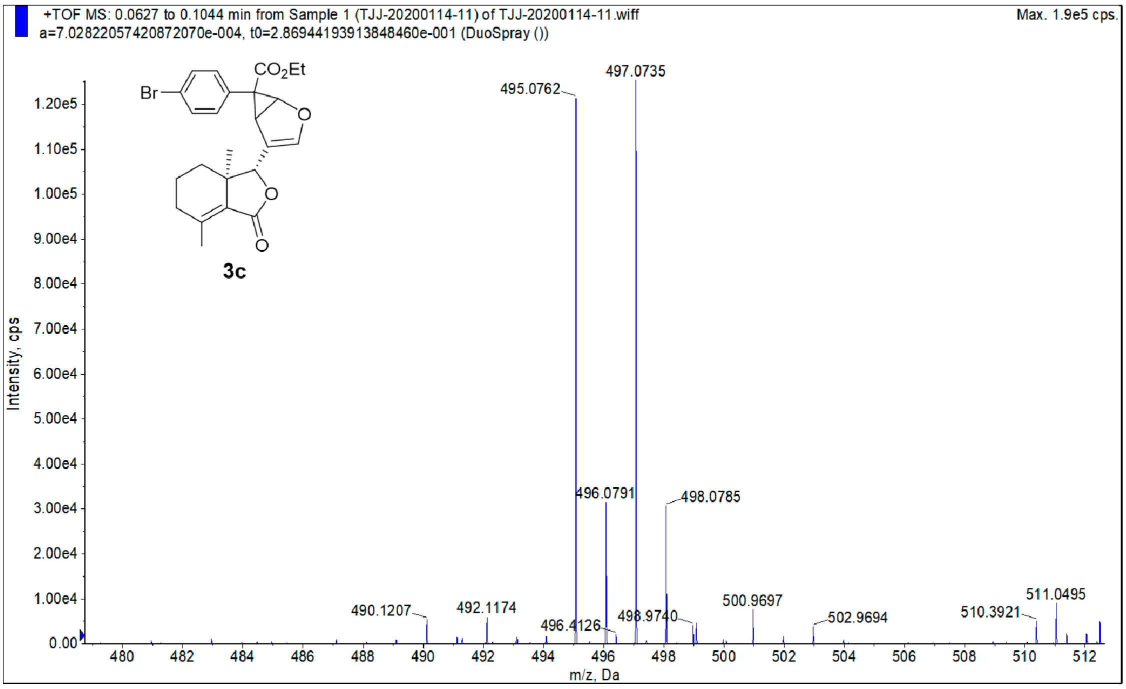

Supplement: Supplementary file 1 [file molecules-25-01109-s001.pdf]
